# The Effect of VPA Treatment on Radiolabeled DOTATATE Uptake: Differences Observed In Vitro and In Vivo

**DOI:** 10.3390/pharmaceutics14010173

**Published:** 2022-01-12

**Authors:** Maria J. Klomp, Leo J. Hofland, Lilian van den Brink, Peter M. van Koetsveld, Fadime Dogan, Corrina M. A. de Ridder, Debra C. Stuurman, Marian C. Clahsen-van Groningen, Marion de Jong, Simone U. Dalm

**Affiliations:** 1 Department of Radiology and Nuclear Medicine, Erasmus MC, 3015 GD Rotterdam, The Netherlands; m.j.klomp@erasmusmc.nl (M.J.K.); l.vandenbrink@erasmusmc.nl (L.v.d.B.); c.deridder@erasmusmc.nl (C.M.A.d.R.); d.stuurman@erasmusmc.nl (D.C.S.); 2 Department of Internal Medicine, Division of Endocrinology, Erasmus MC, 3015 GD Rotterdam, The Netherlands; l.hofland@erasmusmc.nl (L.J.H.); p.vankoetsveld@erasmusmc.nl (P.M.v.K.); f.dogan@erasmusmc.nl (F.D.); 3 Department of Experimental Urology, Erasmus MC, 3015 GD Rotterdam, The Netherlands; 4 Department of Pathology, Erasmus MC, 3015 GD Rotterdam, The Netherlands; m.clahsen-vangroningen@erasmusmc.nl

**Keywords:** peptide receptor radionuclide therapy, PRRT, somatostatin type-2 receptors, SSTR2, valproic acid, VPA, preclinical, histone deacetylase inhibitor, upregulation, epigenetics

## Abstract

**Background:** To improve peptide receptor radionuclide therapy (PRRT), we aimed to enhance the expression of somatostatin type-2 receptors (SSTR2) in vitro and in vivo, using valproic acid (VPA). **Methods:** Human NCI-H69 small-cell lung carcinoma cells were treated with VPA, followed by [^111^In]In-DOTATATE uptake studies, RT-qPCR and immunohistochemistry analysis. Furthermore, NCI-H69 xenografted mice were treated with VPA or vehicle, followed by [^177^Lu]Lu-DOTATATE injection. Biodistribution studies were performed, and tissues were collected for further analysis. **Results:** VPA significantly increased SSTR2 expression in vitro. In animals, a statistically significant increased [^177^Lu]Lu-DOTATATE tumoral uptake was observed when VPA was administered eight hours before [^177^Lu]Lu-DOTATATE administration, but increased tumor SSTR2 expression levels were lacking. The animals also presented significantly higher [^177^Lu]Lu-DOTATATE blood levels, as well as an elevated renal tubular damage score. This suggests that the enhanced tumor uptake was presumably a consequence of the increased radiotracer circulation and the induced kidney damage. **Conclusions:** VPA increases SSTR2 expression in vitro. In vivo, the observed increase in tumoral [^177^Lu]Lu-DOTATATE uptake is not caused by SSTR2 upregulation, but rather by other mechanisms, e.g., an increased [^177^Lu]Lu-DOTATATE circulation time and renal toxicity. However, since both drugs are safely used in humans, the potential of VPA to improve PRRT remains open for investigation.

## 1. Introduction

Peptide receptor radionuclide therapy (PRRT) is an approved and efficient therapy for patients with somatostatin type-2 receptor (SSTR2) positive neuroendocrine tumors (NETs). For PRRT, the radiolabeled somatostatin analogue [^177^Lu]Lu-[DOTA-Tyr^3^]octreotate ([^177^Lu]Lu-DOTATATE) is used, which binds with high affinity to the SSTR2 frequently overexpressed on NETs. Although SSTR2-mediated PRRT improved the progression-free survival of NET patients, complete responses are rare [1,2]. Therefore, research is performed to improve PRRT outcomes via the upregulation of the target receptor SSTR2, using inhibitors which target the epigenetic machinery.

The efficacy of epigenetic drugs to increase SSTR2 expression has already been shown in multiple in vitro studies [3,4,5,6,7,8,9,10,11,12]. Both histone deacetylase inhibitors (HDACis) and DNA methyltransferase inhibitors (DNMTis) have been investigated. Valproic acid (VPA), a HDAC class I inhibitor, is one of the most studied inhibitors. VPA is clinically approved for the treatment of several neurologic disorders, facilitating clinical implementation [13]. Moreover, it was demonstrated in several NET cell lines, e.g., BON-1, NCI-H727 and GOT1, that VPA is superior to other HDACis with regard to SSTR2 upregulation [3,4,5,6]. However, to the best of our knowledge, the effect of VPA on SSTR2 expression and SSTR2-mediated radiotracer uptake has not been studied in vivo yet. 

Therefore, the aim of this study was to examine the effects of VPA on SSTR2 expression levels in vitro and in vivo, using NCI-H69 human small-cell lung carcinoma cells. In vitro, we examined the effect of VPA treatment on SSTR2 expression level and [^111^In]In-DOTATATE uptake, and we investigated whether this effect was time-dependent. Furthermore, we investigated the effect of a single VPA injection on tumor and physiological [^177^Lu]Lu-DOTATATE uptake in NCI-H69 tumor-bearing mice.

## 2. Materials and Methods

### 2.1. Cell Culture

NCI-H69 human small-cell lung carcinoma cells (ECACC) were sub-cultured once a week at a concentration of 180,000 cells/mL (T175 culture flask, 20 mL), and fresh medium was added on day four (30 mL). Cells were cultured up to 20 passages in a humidified atmosphere (37 °C, 5% CO_2_) in RPMI medium 1640 + GlutaMAX-I (Gibco, Breda, The Netherlands) supplemented with 10% (*v*/*v*) fetal bovine serum (Gibco) and 5% (*v*/*v*) penicillin–streptomycin (Merck Life Science NV, Amsterdam, The Netherlands).

### 2.2. Radiolabeling 

DOTATATE (Bachem AG, Budendorf, Switzerland) was radiolabeled with [^111^In]InCl_3_ (Curium Pharma, Petten, The Netherlands) or ^177^Lu (IDB Group, Baarle-Nassau, The Netherlands) with a molar activity between 60 and 200 MBq/nmol, and 50 MBq/nmol, respectively, as previously described [14]. The radiochemical yield was > 95% and radiochemical purity was >90%, measured by thin-layer chromatography and high-performance liquid chromatography, respectively. For internalization studies over time, a mixture of gentisic acid and ascorbic acid (final concentration: 3.5 mM), and ethanol (final concentration: 7%) was added to maintain radiotracer stability (final volume: 140 µL) [14]. [^111^In]In-DOTATATE was used for in vitro experiments, and [^177^Lu]Lu-DOTATATE was used for in vivo studies, thereby taking possible future therapy studies requiring the use of [^177^Lu]Lu-DOTATATE into consideration.

### 2.3. VPA Treatment

The IC_50_ value for VPA (Sigma-Aldrich) was determined based on a seven-day treatment. For this, 30,000 cells were plated in 24-well plates with normal growth medium, and VPA was added after 24 h at a final concentration between 10^−2^ M and 10^−8^ M for dose-response studies or at the IC_50_ dose for other experiments. VPA was dissolved in water, and untreated controls were vehicle-treated (final concentration of 1% H_2_O). For dose–response studies, cell-growth medium and HDACi were refreshed on day four. Cells were then collected by centrifugation on day seven, and the amount of DNA was quantified by using Hoechst 33256 [6]. In short, cells were lysed with 0.2% (*v*/*v*) Triton X-100, and the DNA was sheared by sonification. Subsequently, the DNA concentration was determined after adding Hoechst 33256 and measuring the absorbance at an excitation and emission wavelength of 485 nm and 535 nm, respectively. Fluorescence of experimental samples was referenced to a standard curve of calf thymus DNA.

### 2.4. [^111^In]In-DOTATATE Internalization Studies of VPA-Treated Cells

NCI-H69 cells were treated with VPA up to 48 h with the IC_50_ value to study the time-dependency of treatment. Internalization studies were performed 8, 16, 24, 40 and 48 h after start of VPA treatment as previously described [6], with implementation of minor adjustments. Two million cells were incubated with 1 mL of 1 nM [^111^In]In-DOTATATE (60–200 MBq/nmol), with or without an excess of 1 µM unlabeled DOTATATE in RPMI medium 1640 + GlutaMAX-I supplemented with 1% (*w*/*v*) BSA and 20 mM HEPES (pH 7.4). Cells were incubated at 37 °C for two hours in 1.5 mL Safe-Lock Eppendorf tubes, washed twice with 1 mL cold phosphate-buffered saline (PBS) and subsequently pelleted by centrifugation (5 min, 0.9× *g*, 4 °C). The cell pellets were then measured in the gamma counter to determine the [^111^In]In-DOTATATE uptake. To correct for differences in cell numbers that may occur as a consequence of washing steps, the DNA concentration of three spare samples was determined with Hoechst 33256, according to the protocol described above. These spare samples were also treated with either the vehicle (i.e., 0.1% H_2_O) or HDACi.

### 2.5. RT-qPCR and Immunohistochemistry of VPA-Treated Cells

SSTR2 expression levels were determined 24 h after VPA treatment by using RT-qPCR [6] and immunohistochemistry. For SSTR2 immunohistochemistry, cytospins were made (Rotofix 32A, Hettich, Geldermalsen, The Netherlands) and samples were subsequently fixated with 2% paraformaldehyde (20 min, room temperature (RT)). After permeabilization with 0.1% (*v*/*v*) Triton X-100 in PBS (15 min, RT), samples were blocked with 1% (*w*/*v*) bovine serum albumin (BSA; 1 h, RT). Rabbit SSTR2 antibody (NeoBiotech, Maastricht, The Netherlands, NB-49-015, 1:100 dilution) was added (overnight, 4 °C), and Dako REAL EnVision detection system peroxidase/DAB^+^, rabbit/mouse kit (Agilent Technologies, Amstelveen, The Netherlands) was used as secondary antibody (30 min, RT). Samples were counterstained with hematoxylin, mounted and visualized with the NanoZoomer 2.0 HT (Hamamatsu Photonics, Hamamatsu City, Japan). Afterward, the percentage of cells with positive staining was assessed on three random locations per slide, using a 10x magnification and the CellProfiler software (version 4.0.7), which is freely available to download at www.cellprofiler.org, accessed on 28 November 2021. The percentage of SSTR2 negative cells (Q0) and the extent of SSTR2 expression in the remaining cells were determined. For the latter, vehicle- and VPA-treated cells were pooled together, and the mean total intensity (MTI) of each SSTR2 positive cell was determined. Subsequently, the cells with the minimum and maximum MTI were recorded, and the difference between these two values was divided into four quartiles (Q1–Q4).

### 2.6. Animals

All animal experiments were approved by the Animal Welfare Committee and were in accordance with European law. Male NMRI-Foxn1 nu/nu mice (5–8 weeks, Janvier) were housed under standard laboratory conditions (i.e., aseptic condition and 12 h light/dark cycle), given ad libitum access to water and food and acclimated for a week before the start of the experiment.

### 2.7. Biodistribution Studies

Mice were subcutaneously injected on the right shoulder with NCI-H69 cells: 5 × 10^6^ cells were injected in 100 µL containing 1/3 Matrigel (Corning, Amsterdam, The Netherlands) and 2/3 HBSS. Tumor size was measured twice a week with a caliper, and tumor volume was calculated by using the following formula: π/6 (tumor length * tumor width) ^ (3/2). Animals were divided into eight experimental groups based on body weight and tumor size (Supplementary Materials Table S1). After 18 days, mice were intraperitoneally injected with VPA (200 mg/kg or 400 mg/kg) or vehicle (0.9% NaCl). Four or eight hours after VPA injection, mice were intravenously injected with ~10 MBq/200 pmol/200 µL [^177^Lu]Lu-DOTATATE (50 MBq/nmol) diluted in 1% (*w*/*v*) BSA in PBS (n = 5 per group). To determine the selectivity of [^177^Lu]Lu-DOTATATE uptake, additional groups of mice received ~10 MBq/200 pmol/100 µL [^177^Lu]Lu-DOTATATE (50 MBq/nmol) plus 4000 pmol/100 µL unlabeled DOTATATE intravenously in a single injection four hours after VPA (400 mg/kg) or vehicle (0.9% NaCl) administration (n = 4 per group).

Biodistribution studies were performed four hours after radiotracer injection. All collected organs were weighted and subsequently counted in the gamma-counter (Wallac Wizard 1480 automatic gamma counter; Perkin Elmer, Groningen, The Netherlands). The uptake of radioactivity was expressed as percentage injected dose per gram of tissue (% ID/g). Values were corrected for the amount of radioactivity left in the syringe and at the injection site (tail). For further analysis, several organs were either snap-frozen or formalin-fixed.

### 2.8. RT-qPCR and Immunohistochemistry of Tissue Samples

To measure *SSTR2* mRNA expression levels, snap-frozen tissue was cryosectioned, mixed with lysis buffer and shortly centrifuged. The supernatant was used to determine mRNA expression levels as previously described [6]. For SSTR2 immunohistochemistry, automated IHC, using the Ventana Discovery Ultra (Ventana Medical Systems Inc., Almere, The Netherlands), was used. Formalin-fixed paraffin-embedded sections (4 µm) were stained for SSTR2A, using a universal chromomap DAB detection Kit (#760-159, Ventana). In brief, following deparaffinization and heat-induced antigen retrieval with CC1 (#950-500, Ventana) for 92 min, the tissue samples were incubated with the SSTR2A antibody (Biotrend, Maastricht, The Netherlands, rabbit polyclonal, lot number D19803) in a 1:25 dilution for 60 min at 37 °C. Subsequently, tissue sections were incubated with secondary antibody omnimap anti-rabbit HRP (#760-4311, Ventana) for 20 min, followed by DAB detection. Incubation was followed by hematoxylin II counter-staining (#790-2208, Ventana) for eight minutes, after which tissue sections were exposed to a blue coloring reagent (#790-2037, Ventana) for eight minutes, according to the manufacturer’s instructions. Stained sections were imaged and quantified by using the method described for the in vitro studies.

### 2.9. Renal Tubular Damage

Periodic Acid-Schiff Diastase (PAS+) staining (#860-014,Ventana) was performed by using the Ventana Special Stains Module (Ventana). In brief, formalin-fixed paraffin-embedded sections (3 µm) were deparaffinized and rehydrated by passage through a decreasing ethanol series. Then slides were incubated with PAS diastase for 12 min, followed by several washing steps. PAS periodic acid was added for four minutes at 50 °C, followed by PAS Schiffs for 20 min at 50 °C. Slides were counterstained with PAS hematoxylin for eight minutes. Every section was blindly scored by an experienced nephropathologist (M.C.C.v.G.) for tubular damage (10× magnification), using a 5-point scale, according to the following criteria: tubular dilatation, cast deposition, brush border loss and necrosis. Each parameter was graded in 10 fields with a score of 0–5: 0, no changes; 1, mild, <10%; 2, moderate, 10–25%; 3, severe, 25–50%; 4, very severe, 50–75%; and 5, extensive damage, >75%.

### 2.10. Statistics

In vitro analysis: A spline-LOWESS analysis with point-to-point curve was used to determine the IC_50_ value of VPA. Data for internalization studies and RT-qPCR were normalized to untreated cells and log-transformed. One-way ANOVA with a Tukey’s multiple comparison test was used to detect differences in internalization studies; and to determine differences in RT-qPCR and IHC analysis, a *t*-test was used. All results represent the mean ± SD of at least two independent biological replicates and at least three technical replicates.

In vivo analysis: Biodistribution data (i.e., % ID/g tissue and tumor-to-background ratios) were log-transformed, and a one-way ANOVA with a Dunnett’s multiple comparison test was performed to detect differences between experimental groups. For RT-qPCR results, a *t*-test was used to analyze the normalized and log-transformed data. A one-way ANOVA with a Dunnett’s multiple comparison test was used to detect differences in IHC. All data results represent the mean ± SD.

All statistical analyses were performed by using GraphPad Prism 5 software.

## 3. Results

### 3.1. Effects of VPA on NCI-H69 Cells In Vitro 

**Figure 1:**
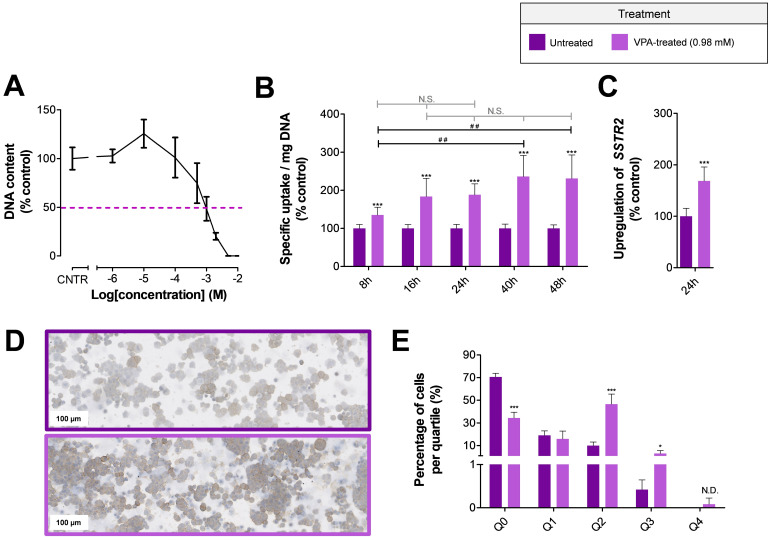
**In vitro effects of VPA on SSTR2 expression** The IC_50_ value of VPA in NCI-H69 cells was 0.98 mM (**A**). To study the effect of VPA on SSTR2 expression, the uptake of [^111^In]In-DOTATATE (per milligram DNA, expressed as percentage of control cells) (**B**), *SSTR2* mRNA expression levels (expressed as percentage of control cells) (**C**) and SSTR2 protein expression levels (**D**,**E**) were examined. The percentage of SSTR2 negative cells (Q0) and the extent of SSTR2 intensity (Q1–Q4) were determined. To indicate significance for *t*-test results between vehicle- and VPA-treated cells, the following abbreviations and symbols were used: * *p* < 0.05; *** *p* < 0.001 and N.D. = non-determined (due to absence of vehicle-treated cells in Q4). For the one-way ANOVA results between different VPA-treated groups, the following abbreviations and symbols were used: *## p* < 0.01 and N.S. = non-significant.

A seven-day cell-growth assay demonstrated that the IC_50_ value for VPA is 0.96 mM in NCI-H69 cells (Figure 1A). Treatment of NCI-H69 cells with the IC_50_ of VPA, followed by uptake studies, showed that the SSTR2-mediated uptake of [^111^In]In-DOTATATE is fast and significantly increased after start of HDACi treatment (Figure 1B). Just eight hours after VPA administration, the uptake of [^111^In]In-DOTATATE was 1.46-fold higher than the uptake measured in untreated cells. No statistical differences were observed between NCI-H69 cells treated with VPA for 16, 24, 40 and 48 h. For practical reasons, it was therefore decided to apply a 24 h VPA-treatment period for further experiments, characterized by a 1.88-fold increased [^111^In]In-DOTATATE uptake compared to untreated cells.

RT-qPCR and immunohistochemical analyses were performed to study *SSTR2* mRNA and SSTR2 protein-expression levels after treating NCI-H69 cells for 24 h with VPA. *SSTR2* mRNA expression levels were significantly upregulated 1.69-fold compared to untreated cells (Figure 1C). In line with this, immunohistochemical analysis showed a clear increase in SSTR2 protein-expression levels (Figure 1D). Quantification of the total SSTR2 staining demonstrated a reduction in the number of SSTR2 negative cells (Q0), i.e., 70.6 ± 3.3% versus 34.4 ± 5.0% for untreated and VPA-treated cells, respectively. Furthermore, a significant increase in the percentage of cells in quartile two (Q2) and three (Q3) was observed after VPA treatment, suggesting an increase in the number of SSTR2-positive cells characterized by higher SSTR2 expression levels (Figure 1E).

### 3.2. Effects of VPA on the Biodistribution of [^177^Lu]Lu-DOTATATE in Mice Bearing NCI-H69 Xenografts 

**Figure 2:**
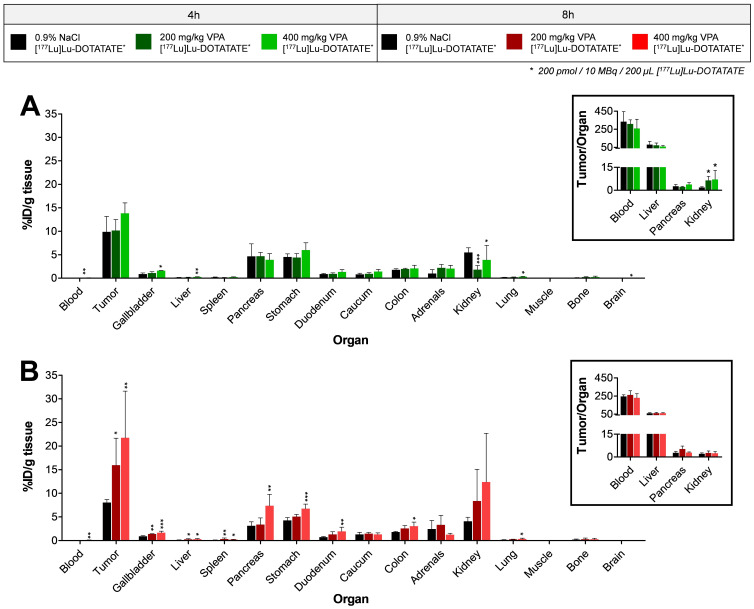
**Biodistribution of [^177^Lu]Lu-DOTATATE after VPA treatment** [^177^Lu]Lu-DOTATATE uptake expressed as percentage injected dose per gram tissue (% ID/g tissue) for animals injected with vehicle or VPA (200 mg/kg or 400 mg/kg) four (**A**) or eight (**B**) hours prior to [^177^Lu]Lu-DOTATATE administration. For both treatment schedules, several tumor-to-organ ratios are depicted: * *p* < 0.05; ** *p* < 0.01; *** *p* < 0.001.

Biodistribution data showed that mice receiving VPA four hours prior to [^177^Lu]Lu-DOTATATE administration had no significant increase in tumor uptake, i.e., 9.9 ± 3.3% ID/g, 10.2 ± 2.3% ID/g and 13.8 ± 2.2% ID/g for untreated, 200 mg/kg and 400 mg/kg VPA, respectively (Figure 2A). In line with this, the uptake of [^177^Lu]Lu-DOTATATE did not change significantly in the majority of background organs of VPA-treated mice in comparison to untreated animals. A significant difference in radioactivity uptake was measured in the kidneys; [^177^Lu]Lu-DOTATATE kidney uptake was lower after VPA treatment (Figure 2A), indicating a faster [^177^Lu]Lu-DOTATATE excretion. Due to this reduction of radioactivity measured in the kidneys, the tumor-to-kidney ratio was significantly increased after VPA treatment, i.e., 1.8 ± 0.5, 6.5 ± 2.6 and 7.1 ± 5.8 for untreated, 200 mg/kg and 400 mg/kg VPA, respectively. In line with what was mentioned above, no significant differences in tumor-to-organ ratio were found for all other organs (Figure 2A and Figure S1A).

To determine whether [^177^Lu]Lu-DOTATATE uptake was SSTR2-mediated after VPA treatment, vehicle or VPA (400 mg/kg) was administered four hours prior to radiotracer injection which was administered with an excess of unlabeled DOTATATE. A less efficient blocking of [^177^Lu]Lu-DOTATATE uptake was demonstrated for VPA-treated mice compared to untreated animals, as shown by significantly increased levels of radioactivity measured in both tumor tissue and background organs of VPA-treated animals (Supplementary Materials Figure S2).

Contrary to the tumor uptake of animals pretreated with VPA four hours before [^177^Lu]Lu-DOTATATE administration, the biodistribution data of mice injected with VPA eight hours before radiotracer injection demonstrated significantly increased [^177^Lu]Lu-DOTATATE tumoral uptake. The tumor uptake was 15.9 ± 5.7% ID/g for 200 mg/kg and 21.7 ± 9.9% ID/g for 400 mg/kg VPA-treated mice compared to 8.0 ± 0.6% ID/g for untreated animals (Figure 2B). However, the physiological uptake of [^177^Lu]Lu-DOTATATE was also significantly increased. Whereas the lower dose of 200 mg/kg VPA only resulted in a statistically significant increased uptake in the gallbladder, liver and spleen, treatment with 400 mg/kg VPA also significantly increased the radiotracer uptake in pancreas, stomach, duodenum, colon and lungs. As a result of the increased physiological [^177^Lu]Lu-DOTATATE uptake and the increased amount of radiotracer in the tumor, no differences in tumor-to-organ ratios were observed (Figure 2B and Supplementary Materials Figure S1B).

Unexpectedly, we observed increased levels of [^177^Lu]Lu-DOTATATE in the blood of VPA-treated mice compared to that of control animals. For mice injected with VPA or vehicle four hours prior to [^177^Lu]Lu-DOTATATE administration, the radioactivity measured in the blood was 0.030 ± 0.001% ID/g, 0.033 ± 0.007% ID/g and 0.062 ± 0.028% ID/g for vehicle, 200 mg/kg and 400 mg/kg VPA-treated mice, respectively. For mice injected eight hours post VPA-administration, this was 0.033 ± 0.004% ID/g, 0.053 ± 0.010% ID/g and 0.085 ± 0.042% ID/g, respectively. The observed increase in radioactivity levels in the blood reached significance for the animals injected with 400 mg/kg VPA. 

### 3.3. Effects of VPA on SSTR2 Expression Levels in Tumor Tissue of NCI-H69 Xenografts

**Figure 3:**
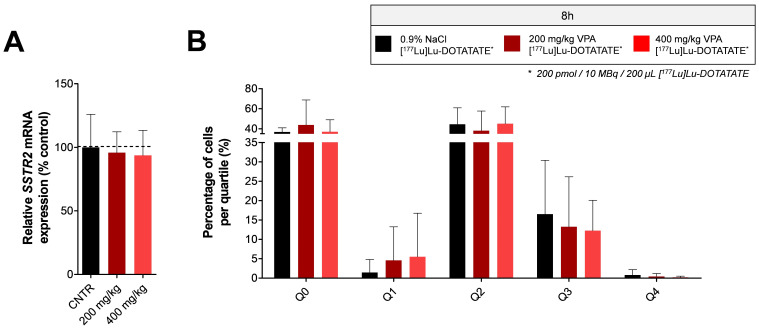
**In vivo tumoral SSTR2 expression levels after VPA-treatment** Tumors of NCI-H69 tumor-bearing mice injected with vehicle or VPA (200 mg/kg or 400 mg/kg) eight hours prior to [^177^Lu]Lu-DOTATATE administration were analyzed for *SSTR2* mRNA expression levels (**A**) and SSTR2 protein-expression levels (**B**). The percentage of SSTR2 negative cells (Q0) and the extent of SSTR2 intensity (Q1–Q4) were determined (**B**).

The increased blood radioactivity levels mentioned above suggest that there is an increased circulation time of [^177^Lu]Lu-DOTATATE. This can potentially result in higher uptake levels of the radiotracer. To determine whether the increased tumor uptake observed in animals injected with VPA eight hours prior to [^177^Lu]Lu-DOTATATE is a consequence of increased SSTR2 expression or a potential effect of prolonged [^177^Lu]Lu-DOTATATE circulation, *SSTR2* mRNA expression levels were measured in tumors of untreated and VPA-treated mice. No statistically significant differences in *SSTR2* mRNA expression levels were observed after VPA treatment (Figure 3A). Immunohistochemistry analysis for SSTR2 expression levels in tumor tissue confirmed the RT-qPCR results, demonstrating no evident increase in SSTR2 protein expression after VPA treatment (Figure 3B). In line with *SSTR2* mRNA expression levels measured in tumor tissue, the expression of *SSTR2* in spleen and liver, both showing an increased [^177^Lu]Lu-DOTATATE uptake after VPA treatment, also showed no significant differences upon treatment (data not shown).

### 3.4. Kidney Tubular Damage

**Figure 4:**
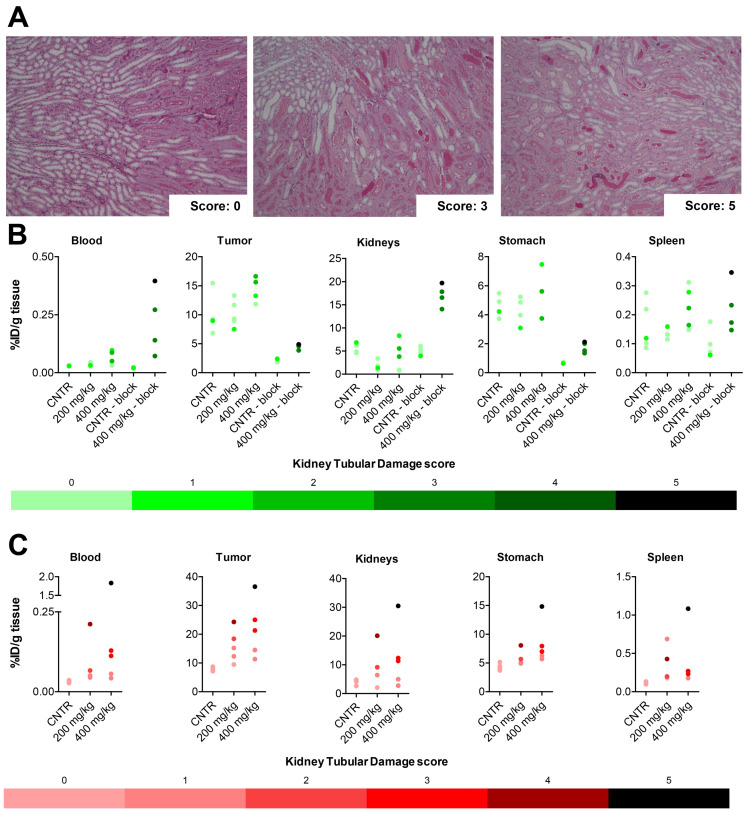
**Biodistribution correlated with the observed renal-tubular-damage** Kidneys were scored for renal tubular damage on a 5-point scale (**A**). Biodistribution data were correlated with the renal tubular damage score for blood, tumor, kidneys, stomach and spleen of mice treated with vehicle or VPA (200 mg/kg or 400 mg/kg) four (**B**) or eight (**C**) hours prior to [^177^Lu]Lu-DOTATATE administration. A color gradient is used to indicate the level of damage. Each data point in these graphs represents a mouse. For the uptake of [^177^Lu]Lu-DOTATATE in kidneys, the average of both kidneys is plotted. Outliers based on % ID/g tissue are included in this figure.

To investigate possible mechanisms behind the increased [^177^Lu]Lu-DOTATATE circulation time, the kidneys were analyzed because of the role of this organ in excretion of the radiotracer. The kidneys were scored for renal tubular damage (Figure 4A). In mice injected with [^177^Lu]Lu-DOTATATE four hours after VPA or vehicle administration, where no significant increase in tumoral [^177^Lu]Lu-DOTATATE uptake was observed, the renal tubular damage was limited, i.e., an average score of 0.2, 0.2 and 1.0 for untreated, 200 mg/kg and 400 mg/kg VPA-treated mice (Table 1). For these three experimental groups, no clear pattern was observed between the kidney damage score and the uptake of [^177^Lu]Lu-DOTATATE (Figure 4B and Supplementary Materials Figure S3). However, 400 mg/kg VPA-treated mice receiving [^177^Lu]Lu-DOTATATE in combination with an excess of unlabeled DOTATATE presented a high average renal tubular damage score of 3.5 (Table 1). In the majority of animals with a high tubular damage score, the uptake of [^177^Lu]Lu-DOTATATE was increased in both tumors and physiological organs when compared to that of animals that were not pretreated with VPA and received an injection of [^177^Lu]Lu-DOTATATE plus an excess of unlabeled DOTATATE (Figure 4B and Supplementary Materials Figure S3).

The kidneys of mice injected with the radiotracer eight hours post-VPA-administration, where a significant increased amount of [^177^Lu]Lu-DOTATATE tumor uptake was observed, were also examined for kidney toxicity. All untreated animals received a damage score of 0, and the damage score for VPA-treated mice varied from 0 to 5. On average, the damage score was 1.6 for treatment with 200 mg/kg and 2.6 for 400 mg/kg VPA. A detailed overview of all damage scores can be found in Table 1. When correlating the renal tubular damage score and [^177^Lu]Lu-DOTATATE uptake of several organs, it was demonstrated that high [^177^Lu]Lu-DOTATATE uptake was often associated with high levels of renal tubular damage (Figure 4C and Supplementary Materials Figure S4).

## 4. Discussion

The low number of complete responses after PRRT in NET patients with SSTR2 positive tumors shows the need for therapy improvement. The aim of this study was, therefore, to examine the effect of the HDACi VPA on SSTR2 expression and radiotracer uptake. To do so, in vitro and in vivo studies were performed by using the SSTR2-expressing NCI-H69 cell line.

The effect of class I-targeting HDACi VPA has been studied before, in vitro, in terms of increased uptake of radiolabeled DOTATATE, *SSTR2* mRNA expression levels and/or SSTR2 protein expression levels, using different NET cell models, i.e., BON-1, QGP1 and NCI-H727 [4,5,7,11,12]. We also studied the effect of VPA and several other HDACis in BON-1, NCI-H727 and GOT1 cells [6], showing the superior effects of VPA. Although convincing effects of VPA on SSTR2 expression levels have been described in vitro, an evaluation of its effect in vivo, including radiotracer uptake, is lacking. 

To examine the effect of VPA in a preclinical setting, we used the human small-cell lung carcinoma cell line NCI-H69. This model has high SSTR2 expression levels, and it is therefore frequently used in studies focusing on PRRT. First, we confirmed that there is an effect of VPA on SSTR2 expression levels in vitro in this model by demonstrating increased [^111^In]In-DOTATATE uptake after VPA treatment. After 24 h, a 1.88-fold increased [^111^In]In-DOTATATE uptake was measured. Moreover, mRNA analysis and immunohistochemistry demonstrated SSTR2 upregulation, thereby excluding other possible mechanisms of action.

It was demonstrated in vitro that the uptake of [^111^In]In-DOTATATE is already significantly enhanced just eight hours after start of VPA treatment. For this reason, we hypothesized that VPA-induced effects arise quickly in vivo as well. This is further supported in the literature, where it is reported that there is an increase in acetylation on histones just two hours after VPA administration [15,16,17]. Moreover, these effects were observed after a single injection. Therefore, we decided to treat animals with a single VPA injection, quickly followed by [^177^Lu]Lu-DOTATATE administration. As we have also demonstrated that effects are quickly reversible in vitro [6], we checked two timeframes between VPA and [^177^Lu]Lu-DOTATATE injection, i.e., four and eight hours. Although we observed significant increased tumor uptake-values in mice treated with VPA eight hours prior to [^177^Lu]Lu-DOTATATE administration, statistically significant increases in tumor-to-organ ratios were lacking. The absence of increased SSTR2 mRNA and protein expression levels, in combination with the increased amount of [^177^Lu]Lu-DOTATATE in the blood of VPA-treated animals, suggested that the enhanced uptake is not caused by SSTR2 upregulation, but most likely by other mechanisms of action, such as an increased [^177^Lu]Lu-DOTATATE circulation time.

To get insights in the possible mechanisms involved in an increased [^177^Lu]Lu-DOTATATE circulation time, the renal tubular damage score was evaluated. It was shown that mice receiving VPA eight hours prior to [^177^Lu]Lu-DOTATATE administration have increased renal damage. This damage most likely causes a slower excretion rate of the radiotracer, resulting in a longer circulation time. However, it remains to be investigated if the increased radiotracer circulation time induces renal damage, or that the renal damage is inducing a prolonged circulation time. Nonetheless, the observed increase in [^177^Lu]Lu-DOTATATE circulation time can lead to an increased radiotracer uptake in both high (e.g., tumor, pancreas and stomach) and low (e.g., liver, spleen and lung) SSTR2-expressing organs. 

Mice pretreated four hours prior to [^177^Lu]Lu-DOTATATE administration did not demonstrate significantly increased [^177^Lu]Lu-DOTATATE tumor uptake. In line with this, the renal tubular damage in this group was still limited. We therefore hypothesize that the extent of renal damage was not progressed enough to influence excretion at this time point. Mice following the same treatment regimen (400 mg/kg VPA, injected four hours prior to [^177^Lu]Lu-DOTATATE administration), but also receiving an excess of unlabeled DOTATATE, are characterized by increased renal tubular damage in comparison to vehicle-treated animals receiving an excess of DOTATATE. We hypothesize that this may be caused by high DOTATATE concentrations damaging the kidneys that are already slightly affected by VPA and [^177^Lu]Lu-DOTATATE combination treatment, radiation-induced nephrotoxicity as a consequence of high kidney-radiation dosages or a combination of these two. The radiation-induced toxicity can be a result of blocking the SSTR2 in the tumor and other physiological organs, leads to higher [^177^Lu]Lu-DOTATATE renal exposure.

The exact mechanism behind the observed renal tubular damage cannot be determined from our data. The observed toxicity can either be a consequence of VPA monotherapy or the combination therapy of VPA and [^177^Lu]Lu-DOTATATE. The observed toxicity cannot be an effect of [^177^Lu]Lu-DOTATATE monotherapy, as vehicle-treated animals receiving only [^177^Lu]Lu-DOTATATE do not demonstrate tubular damage. Furthermore, in a study by Svensson et al. [18], mice were injected with 90, 120 or 150 MBq [^177^Lu]Lu-DOTATATE, which matched an absorbed dose in the renal cortex of 35, 47 and 58 Gy, respectively. The threshold dose value for tubular damage was determined to be approximately 24 Gy. Since animals were administered with only 10 MBq [^177^Lu]Lu-DOTATATE and mice were sacrificed already four hours post-injection of the radiotracer, a kidney dose well below the threshold can be expected in our study, even in VPA-treated animals that received [^177^Lu]Lu-DOTATATE plus an excess of unlabeled DOTATATE associated with a 3–4 times higher kidney uptake. When it comes to VPA, safety issues related to kidney function after a long-term VPA treatment period in rodents have previously been described [19,20,21]. However, to the best of our knowledge, renal toxicity has not been described after administration of a single VPA injection in animals, using the doses applied in our study. Nonetheless, the renal tubular damage of mice treated with VPA only has not been examined in this study, and we can therefore not draw firm conclusions on the toxicity of VPA mono-treatment. Further research on this topic is required. Based on our data, the combination treatment and, thus, the interaction of the two drugs seems to be the most plausible explanation for the renal damage. We observed that animals receiving an excess of unlabeled DOTATATE after VPA treatment had an increased [^177^Lu]Lu-DOTATATE kidney uptake, which, in turn, was associated with a higher kidney-damage score compared to control animals. This higher radioactivity uptake in the kidneys and the herewith higher radiation dose to this organ could potentially lead to radiation-induced kidney damage in animals treated with the combination of [^177^Lu]Lu-DOTATATE and VPA, even though this radiotracer dosage seems safe when the excretion rate is not hampered. Moreover, our data indicate that VPA itself may contribute to the renal toxicity as a higher VPA dosage (400 mg/kg versus 200 mg/kg) or a longer treatment time (8 h versus 4 h) results in a more severe damage. This damage may be aggravated in combination with [^177^Lu]Lu-DOTATATE, especially when a large amount of unlabeled DOTATATE is co-injected. We therefore hypothesize that it is likely that the interaction of VPA and [^177^Lu]Lu-DOTATATE caused the renal toxicity observed in our study.

Even though the effect of VPA in the NCI-H69 cell model did not provide the desired effect in vivo, the potential of SSTR2 upregulation in response to other HDACis or DNMTis in vivo has been described previously. Two articles have been published studying the effect of HDACis by using NET-bearing mice [4,5]. In these two studies, BON-1 and NCI-H727 tumor-bearing mice were treated with FK228 and TDP-A, respectively. After injection of [^68^Ga]Ga-DOTATATE, the PET/CT-scan demonstrated increased standard uptake values after HDACi-treatment, reaching significance for FK228-treated animals. A more elaborative study is performed by Taelman et al. [3], showing statistically significant increased tumor uptake of [^68^Ga]Ga-DOTATOC upon treatment with DNMTi decitabine in BON-1 tumor-bearing mice caused by an increase in SSTR2 protein-expression level. Moreover, the effect of decitabine treatment on [^68^Ga]Ga-DOTATOC on physiological uptake was not significant. These results emphasize the potential value of combining epigenetic drugs and radiolabeled somatostatin analogues. Next to the HDACis used in the studies mentioned above [3,4,5], other HDACis have shown to be promising with regard to SSTR2 upregulation and increased SSTR2-targeting radiotracer uptake in in vitro studies, as was also described in our previous paper [6]. A next step can be to test these HDACis in vivo as well.

We hypothesize that the short half-life of VPA in mice of approximately 55 min may be a major factor preventing VPA from exerting its SSTR2-upregulating capacity [22]. This fast excretion can cause an insufficient tumoral VPA dose. To investigate if an insufficient tumoral dose is the cause of absent effects, VPA can be injected intratumorally. If this is proven to be effective for SSTR2 upregulation, effort could be made to increase the low tumoral VPA dose, e.g., by the use of a constant-rate infusion system, allowing for stable blood concentrations or by applying a tumor-targeting approach. This may support VPA to induce SSTR2 upregulation and, thus, increased [^177^Lu]Lu-DOTATATE uptake. However, the renal damage observed in our study has to be kept in mind, and, in line with this, careful monitoring of potential renal toxicity upon combination treatments, using VPA, is required in preclinical studies, also when VPA is administered differently and/or a lower dose is used. Moreover, although some very rare side effects affecting renal function have been described in humans as well [23], VPA is safely used for the treatment of neurological disorders [13]. Therefore, the potential of this combination therapy to improve PRRT outcomes in humans remains open for investigation.

## 5. Conclusions

In conclusion, VPA induced convincing SSTR2 upregulation in NCI-H69 cells in vitro. Although VPA is frequently studied for SSTR2 upregulation in NET cell lines showing positive results, our preclinical in vivo data demonstrated that a single VPA injection does not result in the desired effect in our mouse model. Although the radiotracer tumor uptake is increased in mice injected with VPA eight hours prior to [^177^Lu]Lu-DOTATATE administration, this is not the result of SSTR2 upregulation, but most likely caused by other mechanisms, such as an increased [^177^Lu]Lu-DOTATATE circulation time and renal toxicity. This observed damage is either the result of VPA monotherapy or, more likely, caused by an interaction between VPA and [^177^Lu]Lu-DOTATATE. The absence of desired effects in vivo may be caused by insufficient tumoral VPA concentrations due to the short half-life of VPA. As higher VPA dosages are not possible due to the observed renal toxicity, VPA is not suitable to increase SSTR2 expression and, thus, PRRT efficacy in this model. However, since VPA rarely causes renal toxicity in humans and shows higher plasma protein binding and longer half-life, the effect of this HDACi on SSTR2 expression, the potentially increased [^177^Lu]Lu-DOTATATE uptake and the herewith associated improved PRRT efficacy remains open for investigation in humans.

## Figures and Tables

**Table 1:** Tubular kidney damage scores (scale: 0–5) given to each experimental group.

	Tubular Kidney Damage Score					
	0	1	2	3	4	5
No VPA (4 h)	4/5	1/5	-	-	-	-
200 mg/kg VPA (4 h)	4/5	1/5	-	-	-	-
400 mg/kg VPA (4 h)	2/5	1/5	2/5	-	-	-
No VPA (4 h, block)	3/4	1/4	-	-	-	-
400 mg/kg VPA (4 h, block)	-	-	-	3/4	-	1/4
No VPA (8 h)	5/5	-	-	-	-	-
200 mg/kg VPA (8 h)	1/5	2/5	1/5	-	1/5	-
400 mg/kg VPA (8 h)	-	2/5	-	2/5	-	1/5

## Data Availability

Data can be requested by contacting the corresponding author.

## Supplementary Materials

The following are available online at https://www.mdpi.com/article/10.3390/pharmaceutics14010173/s1. Table S1: Animal characteristics. Figure S1: Tumor-to-organ ratios. Figure S2: Biodistribution of [^177^Lu]Lu-DOTATATE after VPA treatment in animals co-injected with an excess of unlabeled DOTATATE (block study). Figure S3: Biodistribution data correlated with the observed renal tubular damage (four hours). Figure S4: Biodistribution data correlated with the observed renal tubular damage (eight hours).
