# Supplementary material for: The Effect of VPA Treatment on Radiolabeled DOTATATE Uptake: Differences Observed In Vitro and In Vivo
Source: Pharmaceutics. 2022 Jan 12;14(1):173. doi: 10.3390/pharmaceutics14010173 (PMC8779846; doi:10.3390/pharmaceutics14010173)
Supplement: Supplementary file 1 [file pharmaceutics-14-00173-s001.zip › pharmaceutics-1509059-supplementary-updated.pdf]

pharmaceutics

Supplementary Materials

The Effect of VPA Treatment on Radiolabeled DOTATATE Uptake: Differences Observed In Vitro and In Vivo

Maria J. Klomp, Leo J. Hofland, Lilian van den Brink, Peter M. van Koetsveld, Fadime Dogan, Corrina M.A. de Ridder, Debra C. Stuurman, Marian C. Clahsen-van Groningen, Marion de Jong and Simone U. Dalm

Table S1. – Animal characteristics Number of mice (n), body weight (g) and tumor volume (mm^3) of all experimental groups, measured one day before VPA and [^{177}Lu]Lu-DOTATATE administration. Body weight and tumor volume data are represented as mean ± standard deviation.

	N	Body weight (g)	Tumor volume (mm^3)
No VPA (4 h)	5	34.1 ± 2.6	407.1 ± 201.3
200 mg/kg VPA (4 h)	5	34.5 ± 1.7	419.2 ± 195.0
400 mg/kg VPA (4 h)	5	35.4 ± 1.2	420.1 ± 197.6
No VPA (4 h, block)	4	34.2 ± 2.3	398.2 ± 221.7
400 mg/kg VPA (4 h, block)	4	35.0 ± 2.4	413.7 ± 236.4
No VPA (8 h)	5	35.7 ± 2.3	425.8 ± 237.7
200 mg/kg VPA (8 h)	5	34.1 ± 1.1	419.6 ± 193.4
400 mg/kg VPA (8 h)	5	34.6 ± 2.5	398.1 ± 203.8

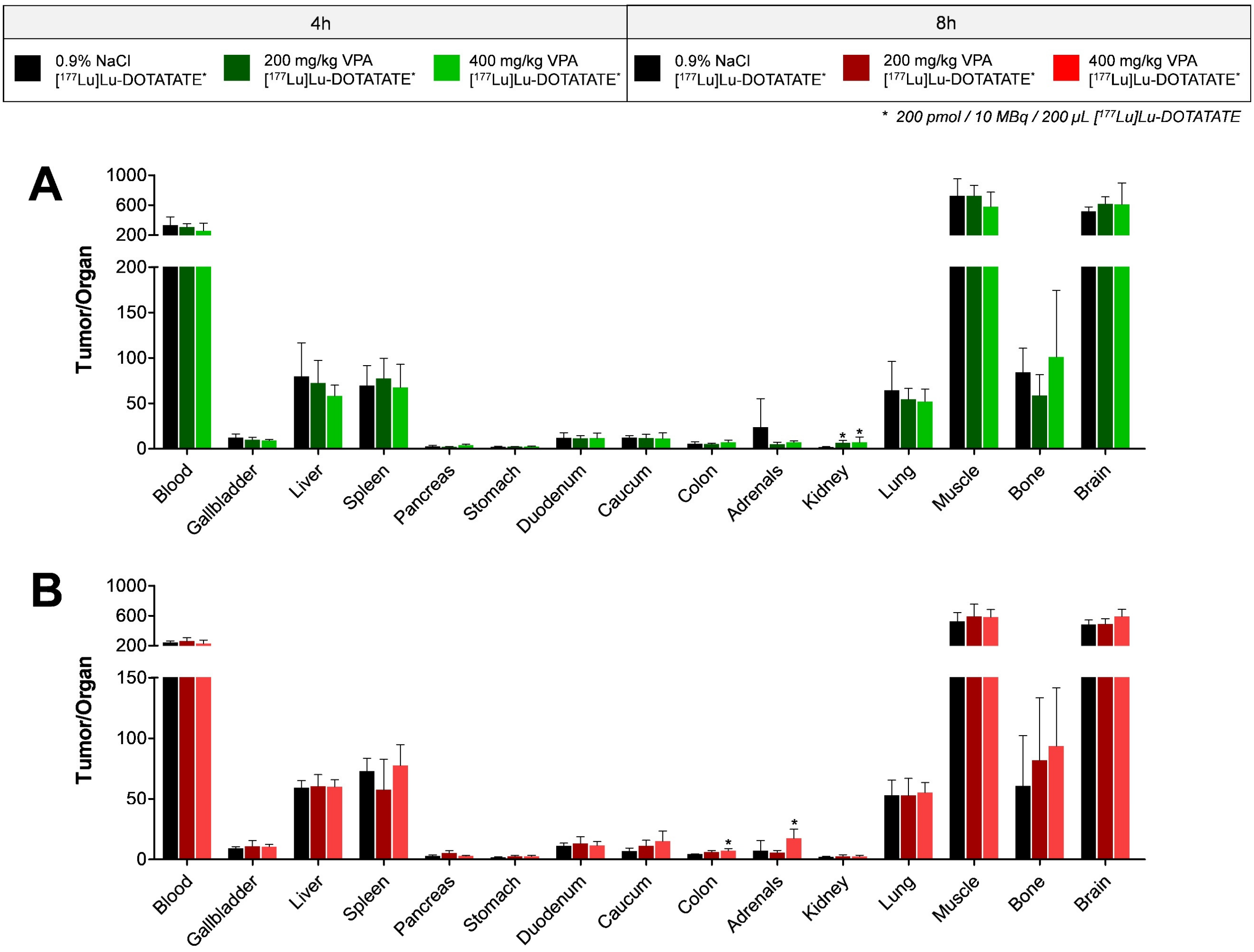

Figure S1. – Tumor-to-organ ratios Tumor-to-organ ratios of mice treated with vehicle or VPA (200 mg/kg or 400 mg/kg) four (A) or eight (B) hours prior to [177Lu]Lu-DOTATATE administration. All data are represented as mean ± standard deviation. * p < 0.05; ** p < 0.01; ***p < 0.001.

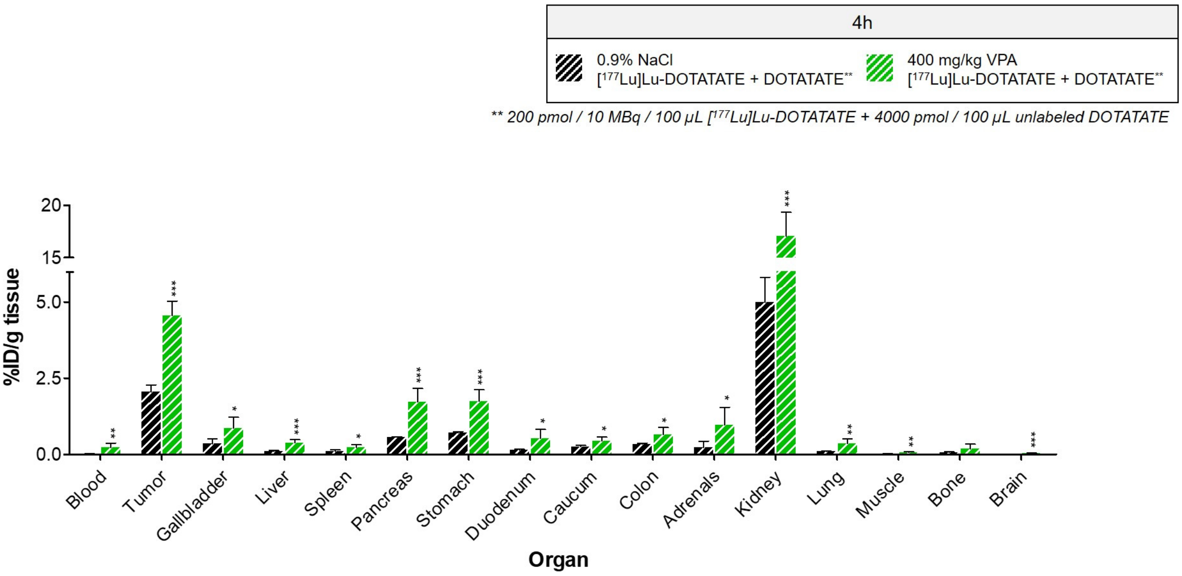

Figure S2. – Biodistribution of [177Lu]Lu-DOTATATE after VPA treatment in animals co-injected with an excess of unlabeled DOTATATE (block study) [177Lu]Lu-DOTATATE uptake expressed as percentage injected dose per gram tissue (%ID/g tissue) for

vehicle- and 400 mg/kg VPA-treated animals injected four hours prior to [^{177}Lu]Lu-DOTATATE administration in presence of an excess of unlabeled DOTATATE. * p < 0.05; ** p < 0.01; *** p < 0.001.

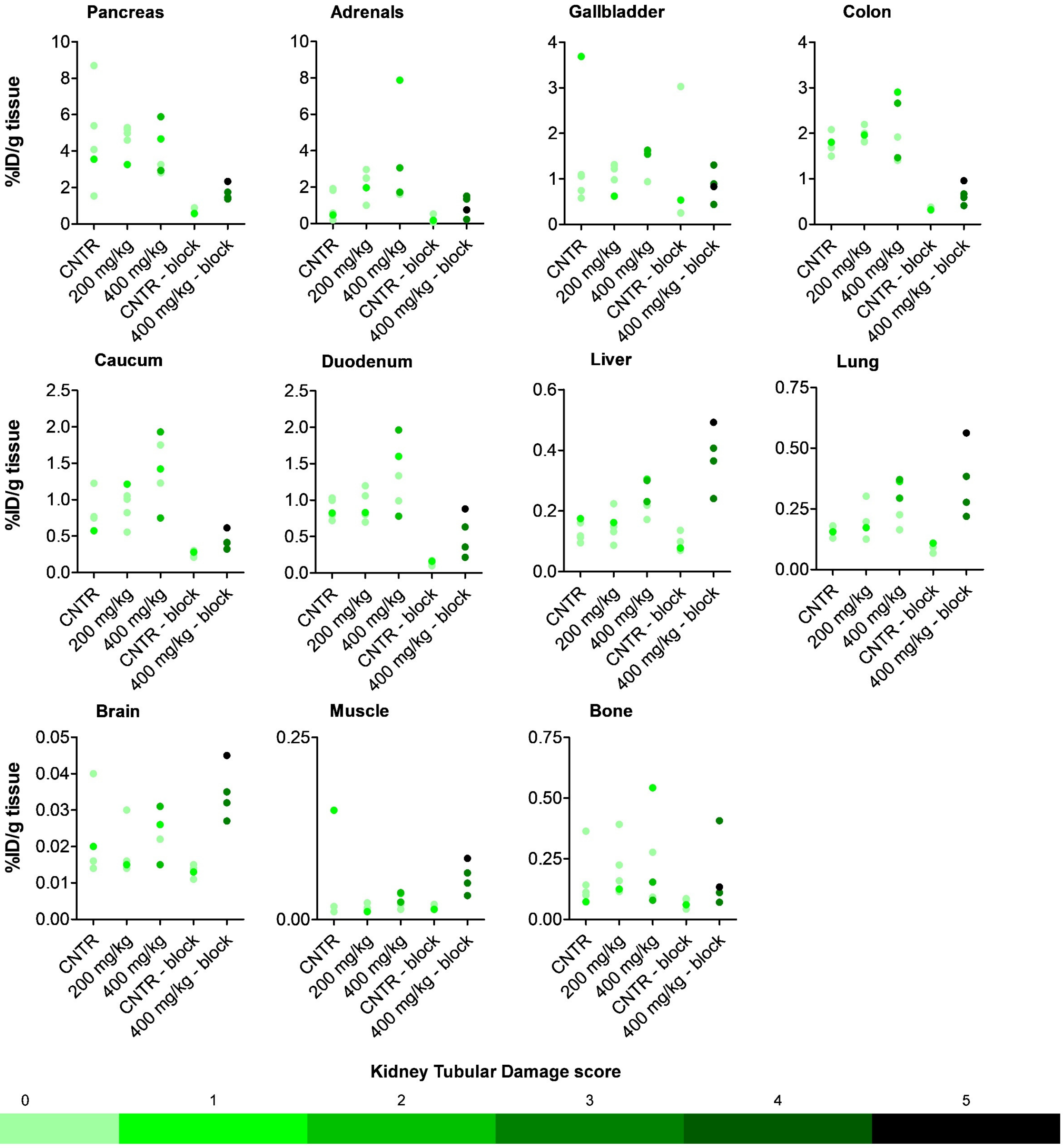

Figure S3. – Biodistribution data correlated with the observed renal tubular damage (four hours) The renal tubular damage score of mice receiving VPA four hours prior to [^{177}Lu]Lu-DOTATATE administration was correlated with the biodistribution data. A color gradient is used to indicate the level of renal tubular damage. Each data point in these graphs represents a mouse. Outliers based on %ID/g tissue are included in this figure.

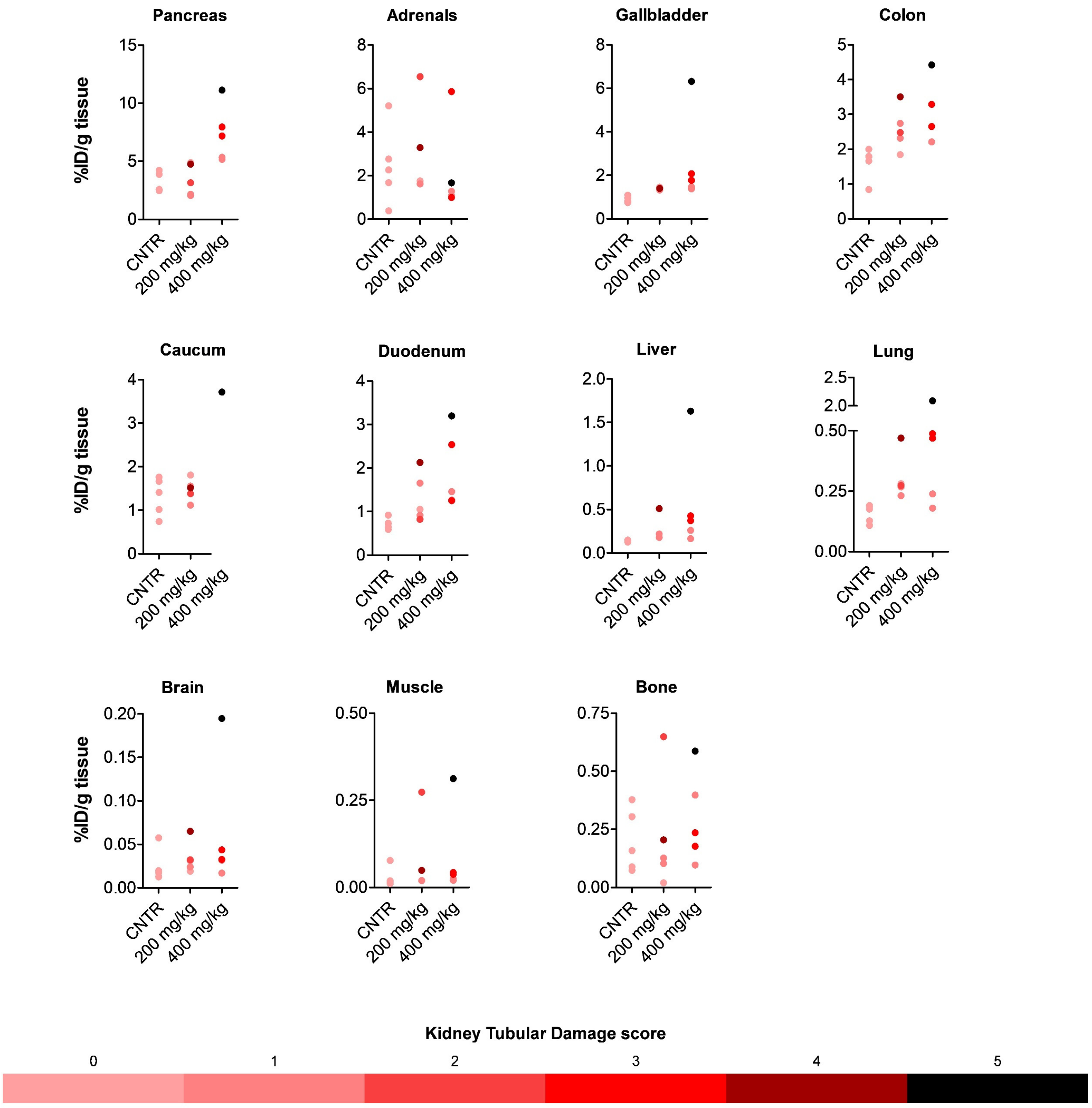

Figure S4. – Biodistribution data correlated with the observed renal tubular damage (eight hours) The renal tubular damage score of mice receiving VPA eight hours prior to [^{177}Lu]Lu-DOTATATE administration was correlated with the biodistribution data. A color gradient is used to indicate the level of renal tubular damage. Each data point in these graphs represents a mouse. Outliers based on %ID/g tissue are included in this figure.
